# Origin of XMRV and its Demise as a Human Pathogen Associated with Chronic Fatigue Syndrome

**DOI:** 10.3390/v3081312

**Published:** 2011-07-27

**Authors:** Oliver Hohn, Norbert Bannert

**Affiliations:** Center for HIV and Retrovirology, Robert Koch Institute, Nordufer 20, 13353 Berlin, Germany; E-Mail: HohnO@rki.de

**Keywords:** XMRV, CFS, origin, recombination

## Abstract

Retroviruses are well known pathogens of mammals, birds and fish. Their potential to induce cancer in chickens was already described almost 100 years ago and murine retroviruses have been a subject of study for 50 years. The first human retroviruses, HTLV and HIV, were discovered more than 30 years ago, surprising researchers and physicians by the profound differences in the diseases they cause. HTLV-1 is able to induce, after decades of infection, lymphomas/leukemia or neuroimmune disorders whereas untreated HIV infection leads almost inevitably to AIDS. The recently described XMRV *(xenotropic murine leukemia virus-related virus)* appeared to possess many of the features known for HTLV and was regarded by some to be the third human retrovirus. However, recent publications by Knox *et al*. [1] and Paprotka *et al*. [2] have shed new light on this gammaretrovirus. Knox and colleagues clearly demonstrate that XMRV is absent in patients belonging to a chronic fatigue syndrome cohort who had previously been reported to be XMRV-positive [3]. This supports the growing suspicion that laboratory contamination was responsible for the postulated link between XMRV and the disease. Furthermore, Paprotka *et al*’s identification of XMRV’s origin and the phylogenetic analysis of known XMRV sequences are further nails in the coffin to the notion that XMRV is a clinically relevant infectious human retrovirus.

## 1. XMRV: A New Human Retrovirus?

XMRV *(xenotropic murine leukemia virus-related virus)* was first reported as a potential new retrovirus of humans in 2006 [4] when a microarray approach revealed its presence predominantly in tissues from patients with familial prostate cancer who were also homozygous for a mutation (R462Q) in the gene for RNASE L, an enzyme involved in innate antiviral immunity. Interestingly, XMRV was initially reported to be present exclusively in stroma cells but not in the tumor cells themselves, a finding that appeared to exclude a common mechanism of malignant transformation by gammaretroviruses. The presence of XMRV in prostate cancer patients was later confirmed by others [5–7], although the virus was usually found in tumor cells and with no correlation to the RNASE L genotype. In sharp contrast, many studies found a much lower prevalence or even complete absence of XMRV [8–14]. One critical result in 2009 seemed to support the link between XMRV and prostate cancer by discovering the virus in the primary prostate carcinoma-derived cell line 22Rv1 [15].

## 2. XMRV and Chronic Fatigue Syndrome

In 2009, attention became more intensely focused on XMRV when Judy Mikovits’ group at the Whittemore Peterson Institute in Reno, Nevada, reported in *Science* that 67% of chronic fatigue syndrome (CFS) patients were XMRV positive [3] and that the virus was also present in the blood of 3.7% of healthy controls. The study used multiple lines of evidence (PCR, virus isolation and detection of specific antibodies) to demonstrate XMRV sequences and proteins in peripheral blood mononuclear cells (PBMC) and the presence of cell-free transmittable virus in patients’ plasma.

This study was immediately challenged by other reports, initially from Europe [16–18] and later from the US [19–22] that could not confirm the findings. The potential association between a new pathogen and CFS and the implied spread of a previously unrecognized gammaretrovirus in humans were nevertheless of great concern. Despite all warnings that the link between CFS and XMRV could not be confirmed, many patients suffering from this debilitating disease and desperately hoping for a cure started taking anti-retroviral drugs. However, the increasing number of reports failing to find a retroviral etiology for CFS and other disorders [23–26] cast further doubt on the reliability of the original findings. The authors and enthusiasts of the original CFS study [3] attempted to dismiss such negative results on the grounds of inadequate diagnostic procedures or inappropriate selection of patients [27], demanding that the methods used in the original paper be meticulously replicated. However, a recent study [28] again found no evidence for XMRV despite using virtually identical PCR, serology and virus isolation methodologies to investigate samples from some of the same CFS patients found to be positive in the initial study [3].

The prevalence of XMRV in CFS patients was also addressed by Knox and colleagues [1] who investigated a total of 61 CFS patients of which 43 had previously been found to be XMRV-positive by a commercial (VIPDx, Reno, NV, USA) or research laboratory (Whittemore Peterson Institute, Reno, NV, USA) using the methodology of Lombardi *et al*. [3]. Duplicate blood samples drawn on the same day by the same phlebotomist were available for 19 of these patients and, remarkably, in 10/19 cases samples tested positive for XMRV DNA by VIPDx while 0/19 tested positive by Knox *et al*. [1]. To broaden the study, samples from a subset of 29 CFS patients were subjected to a barrage of different tests: PCR and RT-PCR on nucleic acids from stimulated or unstimulated PBMC, RT-PCR for the detection of viral RNA in the plasma and even incubation of susceptible mink lung cells with patients’ plasma or PBMC culture supernatants to facilitate enhanced detection of infectious virus particles. Despite 26 of these 29 CFS patients having been previously tested positive by VIPDx or the Whittemore Peterson Institute using one or more of these methods, no trace of XMRV could be detected. Furthermore, when Knox *et al.* investigated 60 plasma samples for the presence of XMRV-specific antibodies using an established chemiluminescence immunoassay [1], just one was weakly reactive for XMRV gp70 and the specificity of this reaction could not be confirmed by Western blot.

## 3. The Origin of XMRV

Paprotka and colleagues [2] decided to track down the origin of XMRV. As mentioned above, the prostate cancer cell line 22Rv1 contains over 10 proviral XMRV copies/cell [15]. This cell line was developed by the serial passage of a human prostate xenograft (CWR22) in nude mice from 1992 to 1999 and it was therefore important to know whether the virus was already present in the patient who provided the xenograft or if XMRV infection of the human cells occurred during passaging in mice.

**Figure 1:**
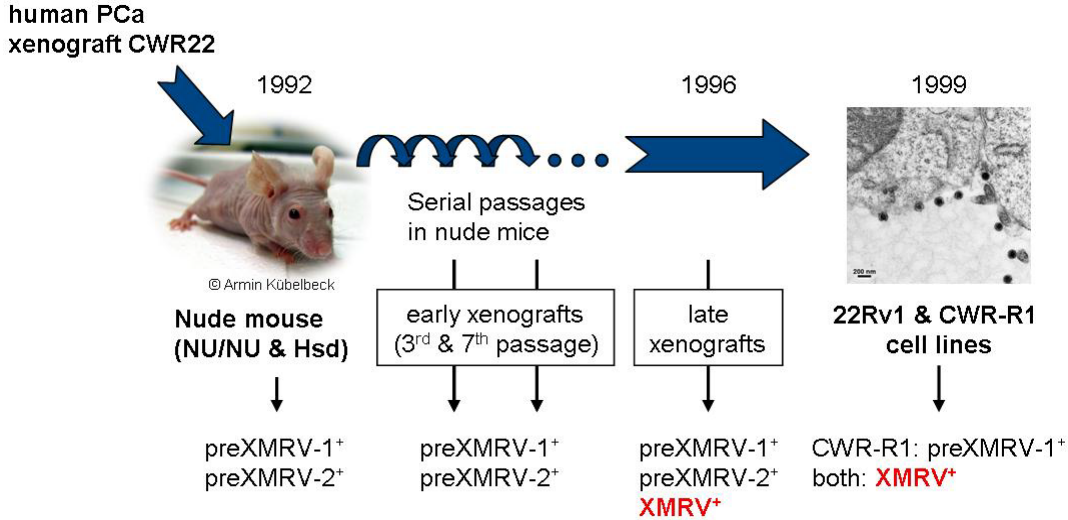
Generation of XMRV during passage of the CWR22 xenograft [2]. Human prostate cancer tissue was xenografted into nude mice (that carry both endogenous proviruses parental to XMRV) in 1992. Due to the presence of varying traces of murine cells, all xenografts and the CWR-R1 cells were positive for preXMRV-1 and in some cases also for preXMRV-2. XMRV is only present in late xenografts generated after 1996 and in both of the derived cell lines. (Nude mouse picture by A. Kübelbeck [30]).

Paprotka and colleagues therefore characterized XMRV infection in early and late passages of the prostate cancer xenograft that eventually gave rise to the cell lines 22Rv1 and CWR-R1. They found no evidence for ‘human’ XMRV in early passages of the tumor xenograft but found instead two XMRV-related proviruses with complementary regions of high homology to prototypic XMRV, sequences that they named preXMRV-1 and preXMRV-2. However, later passages of the xenograft and both resulting cell lines contained the XMRV prototype sequence closely matching that reported in humans (Figure 1). This clearly demonstrates that the virus was not present in the original human tumor but was acquired later.

**Figure 2:**
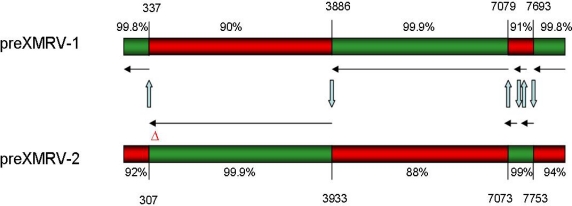
Recombinant origin of the XMRV sequence [2]. The preXMRV-1 and preXMRV-2 genomes with their homology to the XMRV consensus sequence are shown. Template switching events indicated with blue arrows occurred in regions of 20–73 nucleotide identity between both viral RNAs. Nucleotide numbers refer to 22Rv1 XMRV (Acc. No. FN692043). Six template switching events result in a sequence differing from all known XMRV isolates by only 4–13 nucleotides.

What, then, was the source of preXMRV-1 and preXMRV-2 in the xenografts and where did ‘human’ XMRV form? By the time of testing, the xenografts had presumably become infiltrated with murine cells and the preXMRV-1 and preXMRV-2 proviruses were therefore amplified from traces of genomic murine DNA in the xenograft samples. This was confirmed by analyzing tissues from the strains of nude mice likely to have been used in the 1992–1999 xenograft experiments. The complementary XMRV precursor proviruses (preXMRV-1 and -2) were indeed found in two of these strains (but no ‘human’ XMRV) and the authors therefore concluded that in the mid-1990s recombination between the two parental MLVs in a xenografted nude mouse gave birth to XMRV. While the replication-defective preXMRV-1 is closely related to xenotropic endogenous MLV, pre-XMRV-2 resembles the polytropic or modified-polytropic type. The fact that preXMRV-1 (xenotropic) contributed the envelope protein to ‘human’ XMRV might explain why the new virus could replicate in the human xenograft but not in the murine host itself. Comparing the sequences of preXMRV-1 and preXMRV-2 with that of XMRV, the authors suggest that a virus particle containing both preXMRV-1 and preXMRV-2 genomic RNA must have formed. This particle then infected a cell and six reverse transcriptase template switching events during minus-strand DNA synthesis resulted in the mosaic sequence known as XMRV (Figure 2). This scenario is completely plausible as it is known that the RNAs of two related proviruses can occasionally be incorporated into a single virion and that reverse transcriptase usually switches between the two RNA templates an average of four times per cycle [29].

Paprotka *et al*. calculated that the likelihood of precisely this recombination pattern occurring independently a second time to create the same XMRV sequence to be extremely low, *i.e*., only 1.3 × 10^−12^. As every XMRV sequence published to date has the same six predicted recombination sites, it is clear that they all originate from this unique recombination event during the establishment of the 22Rv1 cell line. Indeed, the possibility of XMRV having a recombinant origin had already been suggested [31]. XMRV itself appears to be completely absent in mice: none of the 45 laboratory and 44 feral mouse strains tested by the authors harbored the complete provirus. However, some have one or other of the parental precursor viruses and both are found simultaneously in just two strains (those used for passage of the original xenograft). This general lack of XMRV in mice virtually rules out the possibility that mice have passed the virus onto humans.

## 4. Concluding Remarks

Although rhesus monkeys can be infected with very high amounts of XMRV [32], there is substantial evidence that the virus does not easily infect, replicate and circulate in humans. The first indication is that XMRV, like other xenotropic MLVs, is subject to a severe restriction of replication in primary human cells through the activity of antiviral factors such as APOBEC3 and tetherin [33–35]. These protective proteins inhibit efficient replication in human PBMC and most likely in other primary human cells. We and others have demonstrated that PBMC have a low susceptibility to XMRV infection and, if infected, release very low levels of infectious virus [26,36].

Furthermore, Knox and colleagues [1] showed that XMRV and other xenotropic MLVs are inactivated by sera from both healthy donors and CFS patients, probably by the action of human complement. Finally, many groups, including that of Knox *et al*, have demonstrated the high probability of false-positive PCR results being the reason for the earlier reports linking XMRV to disease [1,20,28,37,38]. Contamination of patient samples, cells in culture or lab reagents with murine DNA, plasmid DNA containing XMRV sequences or even the virus itself [39] can easily occur if stringent precautions are not taken. Finally, the lack of variation between sequences supposedly obtained from different individuals [1,40] argues against the presence of XMRV in humans as this is not consistent with our current knowledge of retrovirus replication and spread.

In summary, taking all data and the many negative studies into account, there is little to support the idea that XMRV is a retrovirus infecting humans and that it has clinical relevance for prostate cancer or CFS. It transpires that XMRV is not a new human tumor virus after all, but rather the latest example of a “rumor” virus [41].
